# Renal recovery after acute kidney injury requiring dialysis: Predictors and long-term outcomes

**DOI:** 10.5339/qmj.2025.124

**Published:** 2025-12-15

**Authors:** Mostafa Elshirbeny, Mohamed Amin, Rasha Abdulrahman, Tarek Ghonimi, Iman Khater, Ayman Al-Dahshan, Abdullah Hamad, Fadwa Al-Ali, Hassan Almalki, Mohamad Alkadi

**Affiliations:** 1 Nephrology Division, Medicine Department, Hamad Medical Corporation, Doha, Qatar; 2 Preventive Medicine Division, Medicine Department, Hamad Medical Corporation, Doha, Qatar *Email: melshirbeny@hamad.qa

**Keywords:** Acute kidney injury, dialysis, renal recovery, end-stage kidney disease

## Abstract

**Background:**

Acute kidney injury requiring dialysis (AKI-D) is a severe medical condition that is common and associated with a high rate of morbidity and mortality. Identifying predictors of kidney recovery in patients with AKI-D might lead to better care and improved kidney and patient survival. This study aims to assess the long-term clinical outcomes of patients who had AKI-D during their hospitalization and remained on dialysis at discharge and identify predictors of renal recovery after discharge.

**Methods:**

We retrospectively studied adult patients hospitalized between January 2016 and December 2022 who had AKI-D during their hospitalization and continued receiving dialysis after discharge. Patients who had less than three months of follow-up, underwent kidney transplantation, or died within three months of dialysis initiation were excluded from the study.

**Results:**

Of the 64 patients in the study, 20 (31%) achieved renal recovery, while 44 (69%) remained dialysis dependent. The average time to renal recovery was 93 ± 61 days. Recovered AKI-D patients had significantly lower baseline and average weekly predialysis serum creatinine after discharge and significantly higher intensive care unit admission, length of hospital stay, vasopressor use, and number of dialysis sessions than non-recovered patients. Using multivariate analysis, we identified vasopressor use as the only independent predictor of renal recovery after discharge in patients with AKI-D (odds ratio, 16.244 [95% CI, 1.22–217.17]; *P* = 0.035).

**Conclusion:**

Renal recovery after discharge can be seen in up to one-third of patients with AKI-D, even if they have advanced chronic kidney disease at baseline or require dialysis for more than three months. The chance of renal recovery is higher in patients who require vasopressor use during hospitalization. Thus, patients with AKI-D should be closely monitored after discharge, and guidelines on managing such patients need to be created.

## 1. INTRODUCTION

Acute kidney injury requiring dialysis (AKI-D) is a common and severe condition associated with significant morbidity, mortality, and progression of chronic kidney disease (CKD).^1^ AKI-D occurs in approximately 1% to 2% of hospitalized patients and 6% to 7% of critically ill patients, with hospital mortality rates approaching 50%.^2^

The outcomes of AKI-D generally include recovery of kidney function without dialysis, continued dialysis until end-stage kidney disease (ESKD), or death due to renal or non-renal causes. Although up to 90% of all AKI cases may recover kidney function, fewer than 60% of AKI-D patients regain dialysis independence.^3^ The timing and extent of recovery are critical—non-recovery at discharge is associated with worse outcomes than either early or late recovery.^4–6^ Despite the potential benefits of renal recovery, clinical guidelines remain limited, and effective therapies are lacking. Therefore, identifying strategies that promote kidney recovery in AKI-D is urgently needed, as they might improve both renal and overall outcomes.^7,8^

Most studies on patients with severe acute kidney injury (AKI) have focused on the inpatient setting, with limited attention to patients who survive hospitalization but remain dialysis-dependent at discharge. Only a few studies have explored risk factors that might affect renal recovery in AKI-D cases after hospitalization, such as congestive heart failure, frequency of intradialytic hypotensive episodes, fluid removal volume, and preexisting renal impairment.^9–14^ Further studies are warranted to determine long-term clinical outcomes and predictors of renal recovery in outpatient settings, with emphasis on modifiable factors that could pave the way for developing clear guidelines for the outpatient management of AKI-D patients.

Although renal replacement therapy (RRT) can be life-saving in severe AKI, it is often accompanied by hemodynamic instability.^15^ Vasopressors and inotropes are frequently administered to manage this instability; however, their impact on outcomes after RRT initiation remains unclear. In AKI patients requiring RRT, assessing the effect of specific agents is challenging, as their pharmacokinetics are altered by kidney dysfunction and critical illness. Moreover, catecholamines such as norepinephrine, epinephrine, and dopamine have short plasma half-lives, high non-renal clearance, and limited extracorporeal removal, making their clinical impact during RRT complex.

We conducted this study to assess the long-term clinical outcomes of patients who had AKI-D during hospitalization and remained dialysis-dependent at discharge and to identify predictors of renal recovery post-hospital discharge.

## 2. METHODS

### 2.1 Study design and patient population

**Figure 1:**
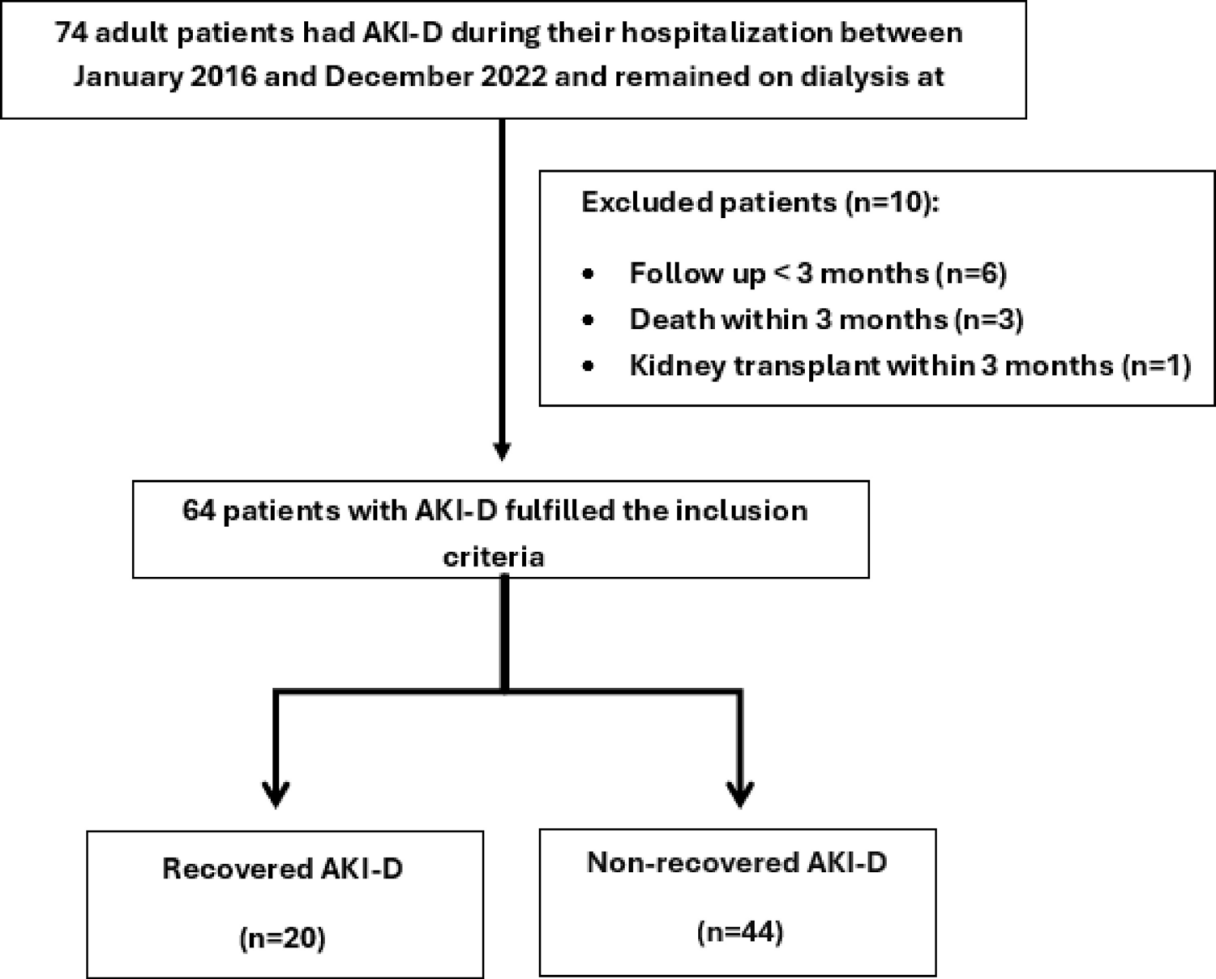
Study design of patients who had AKI-D during hospitalization and remained dialysis-dependent at discharge.

This single-center retrospective study included adult patients (aged ≥18 years) who were admitted to Hamad General Hospital in Doha, Qatar between January 1, 2016, and December 31, 2022, developed AKI-D during hospitalization, and continued to require hemodialysis after discharge at the Fahad Bin Jassim Kidney Center (FBJKC)—the largest dialysis unit in Qatar, with 92 dialysis stations. Patients were excluded if they were under 18 years of age, had less than 3 months of follow-up, died, or underwent kidney transplantation within three months of initiating dialysis. An overview of the study design is provided in Figure 1.

A structured follow-up program for post-discharge AKI-D patients for 6 months was initiated at FBJKC in 2016. All patients had weekly assessment by a nephrologist and dialysis nurse with monitoring of medications (adequate dosing and avoiding nephrotoxic drugs), clinical signs (blood pressure and volume status), blood tests (kidney function and serum electrolytes), and weekly 24-hour urine collection for six months from the initiation of dialysis.

Renal recovery following AKI-D was defined as achieving dialysis independence for a minimum of four weeks. Patients who recovered renal function were subsequently referred, based on their estimated glomerular filtration rate (eGFR) calculated using the CKD Epidemiology Collaboration (CKD-EPI) equation, to either a low clearance clinic (eGFR < 25 mL/min/1.73 m^2^) or a general nephrology clinic (eGFR ≥ 25 mL/min/1.73 m^2^). These patients were followed until December 2022, or until they resumed dialysis, underwent kidney transplantation, left the country, or died—whichever occurred first.

### 2.2 Data collection

We collected data from the national electronic medical record system (Cerner, North Kansas City, MO). Data included demographics (age, sex, and race), comorbidities (diabetes, hypertension, congestive heart failure, peripheral vascular disease, coronary artery disease, active cancer, liver cirrhosis, and CKD), intensive care unit (ICU) admission, admission labs (serum creatinine, urea, albumin, and hemoglobin), cause of AKI, modality of RRT (intermittent hemodialysis or continuous RRT), mechanical ventilation, vasopressors use, number of days of inpatient RRT, vascular access, serum creatinine, urea, albumin, and hemoglobin at the time of discharge. We also collected data post-discharge, such as the number of hemodialysis sessions per week, pre- and post-dialysis mean arterial pressure, interdialytic weight gain, number of intradialytic hypotensive episodes (defined as a systolic blood pressure less than 90 mm Hg), weekly predialysis serum creatinine, urea, hemoglobin, and nutritional parameters, and hospital readmission. We determined the baseline eGFR using the CKD-EPI equation based on the lowest stable serum creatinine within one year before hospitalization.

### 2.3 Statistical analysis

Continuous variables were presented as mean ± standard deviation, and categorical variables were expressed as frequencies and percentages. Comparisons between patients who recovered renal function and those who did not performed using the paired *t*-test for continuous variables and the chi-square test for categorical variables. Variables with a *P* value <0.05 in univariate analyses were entered into a multivariable logistic regression model to identify independent predictors of renal recovery following AKI-D. A *P* value <0.05 in the multivariable analysis was considered statistically significant. All analyses were performed using the Statistical Package for the Social Sciences version 17.0 for Windows (SPSS Inc., Chicago, IL).

## 3. RESULTS

### 3.1 Characteristics of the study population

Between January 2016 and December 2022, 74 patients had AKI-D during hospitalization at Hamad General Hospita (HGH) and continued to receive dialysis at FBJKC after discharge. Sixty-four patients fulfilled the inclusion criteria and were analyzed in the study (Figure 1). All patients underwent hemodialysis, and none required continuous RRT or peritoneal dialysis. The mean age of patients was 64.3 ± 15.33 years, and 54.6% were female. Almost all patients with AKI-D were Middle Eastern (95%). The main comorbidities were CKD (85.9%), hypertension (87.5%), and diabetes (81.3%). Regarding baseline kidney function, seven patients (10.9%) had an eGFR ≥ 60 mL/min/1.73 m^2^, 11 patients (17.2%) had an eGFR between 30 and 59 mL/min/1.73 m^2^, 25 patients (39.1%) had an eGFR between 15 and 29 mL/min/1.73 m^2^ and 19 patients (29.7%) had an eGFR <15 mL/min/1.73 m^2^. Two patients (3.1%) had no documented baseline kidney function within 1 year before hospitalization; for these patients, the eGFR obtained within the 2 years preceding hospitalization was used. Thirty-nine percent of patients were admitted to the ICU (*n* = 25); 22% required vasopressors (*n* = 14), and 16% required mechanical ventilation (*n* = 10). The mean baseline creatinine was 271.2 ± 138.9 μmol/L (3.07 ± 1.57 mg/dL), and the mean baseline eGFR was 29.4 ± 26 mL/min/1.73 m^2^. On admission, the mean serum creatinine was 583.6 ± 238.4 μmol/L (6.60 ± 2.70 mg/dL). Acute tubular injury (ATI) was the most common cause of AKI-D (50%), followed by cardiorenal syndrome (18.8%). The average number of days on RRT was 25.9 ± 23.0, and the average number of hemodialysis sessions during hospitalization was 12.5 ± 11.4. Central venous catheter was the primary vascular access used in RRT (93.8%), while 6.3% of patients (*n* = 4) used their matured arteriovenous fistula (AVF). Patients’ baseline characteristics are summarized in Table 1.

### 3.2 Clinical outcomes of the study population

We prospectively followed all patients with AKI requiring dialysis (AKI-D) for 6 months from the initiation of hemodialysis during their index hospitalization. During the follow-up period, 27 patients (42.2%) were readmitted to the hospital. At 6 months, renal recovery—defined as dialysis independence—was achieved in approximately one-third of patients (*n* = 20), while 44 patients (69%) remained dialysis-dependent. Patients with a baseline eGFR ≥60 mL/min/1.73 m^2^ demonstrated the highest renal recovery rate (57%), whereas those with a baseline eGFR <15 mL/min/1.73 m^2^ had the lowest recovery rate (21%). However, this difference was not statistically significant (*P* = 0.15). The mean time to renal recovery was 93 ± 61 days. Among those who recovered, 20% (*n* = 4) did so within the first month after initiating dialysis, 30% (*n* = 6) recovered between one and 3 months, and the remaining 50% (*n* = 10) regained kidney function after more than three months. A summary of patient outcomes is presented in Table 2.

### 3.3 Predictors of renal recovery in patients with AKI-D

Out of the 64 patients with AKI-D, 20 (31%) had renal recovery and became dialysis-independent, while 44 (69%) remained dialysis-dependent. There were three non-Middle Eastern patients in the AKI-D recovered patients, while all non-recovered patients were from the Middle East (*P* = 0.03). Compared to non-recovered patients, recovered patients had significantly lower baseline serum creatinine (210.1 ± 128.7 μmol/L vs. 293.1 ± 139.6 μmol/L; *P* = 0.03) and, after discharge, had a significantly lower average weekly predialysis serum creatinine (334.0 ± 162.7 μmol/L vs. 491.8 ± 186.2 μmol/L; *P* = 0.002). ICU admission (60% vs. 30%; *P* = 0.03), vasopressor use (50% vs. 9%; *P* < 0.001), length of hospital stays (39.0 ± 33.7 vs. 20.8 ± 13.3; *P* = 0.003), and number of hemodialysis sessions during hospitalization (18.4 ± 17.4 vs. 10.2 ± 5.9; *P* = 0.006) were significantly higher in recovered than non-recovered patients, respectively. The two groups had no significant differences regarding age, gender, comorbidities, cause of AKI, dialysis access, blood urea nitrogen at hemodialysis initiation and discharge, or intradialytic hypotension (Table 3). Using multivariate analysis, we identified vasopressor use as the only independent predictor of renal recovery in AKI-D patients after discharge (odds ratio, 16.244 [95% CI, 1.22–217.17]; *P* = 0.035; Table 4).

### 3.4 Vasopressor use and renal recovery

Fourteen AKI-D patients required vasopressors (10 in the recovered group and 4 in the non-recovered group). Dopamine and norepinephrine were the most commonly used agents (Supplementary Table 1). Dopamine was more frequently used in the recovered group (70% vs. 25%), whereas norepinephrine was more frequently used in the non-recovered group (100% vs. 40%); however, these differences were not statistically significant (*P* = 0.24 and *P* = 0.085, respectively). A higher proportion of recovered patients required more than one vasopressor compared with non-recovered patients (60% vs. 25%), but this difference did not reach significance (*P* = 0.56). The average duration of vasopressor use was similar between recovered and non-recovered groups (3.6 ± 2.9 vs. 7.0 ± 6.9 days; *P* = 0.11; Table 5).

### 3.5 Long-term outcomes of recovered AKI-D patients

AKI-D patients who achieved renal recovery and became dialysis-independent were referred for outpatient follow-up to either nephrology clinics (for those with eGFR ≥ 25 mL/min/1.73 m^2^) or low-clearance clinics (for those with eGFR < 25 mL/min/1.73 m^2^). These patients were monitored until December 31, 2022, with a mean follow-up duration of 17.6 months. Among the 20 patients who recovered, seven (35%) died without requiring dialysis reinitiation, with a mean duration of 14.86 ± 12.10 months from the time of renal recovery. Five patients (25%) resumed dialysis after an average of 18.8 ± 15.1 months. One patient underwent kidney transplantation seven months following recovery. Three patients (15%) continued regular follow-up in clinic for 5 to 49 months post-recovery, while the remaining four patients left the country between 7 and 44 months after becoming dialysis independent. The long-term outcomes and corresponding eGFR values of these patients are detailed in Supplementary Table 2.

## 4. DISCUSSION

In this retrospective study, we reviewed 64 patients who developed AKI requiring dialysis (AKI-D) during hospitalization and remained on dialysis following discharge. At 6 months from the initiation of hemodialysis, approximately one-third of patients (*n* = 20) achieved renal recovery and became dialysis-independent, consistent with previously reported recovery rates ranging from 20% to 45%.^9–14^ However, in contrast to earlier studies, we did not observe a significant association between pre-existing CKD and renal recovery. This discrepancy may be attributed to the high prevalence of CKD in our cohort, with a relatively low average baseline eGFR (mean, 29.4 ± 26 mL/min/1.73 m^2^); notably, 69% of patients had a baseline eGFR < 30 mL/min/1.73 m^2^. Additionally, we found no significant impact of other comorbidities on the likelihood of renal recovery.

Previous studies have shown that critically ill AKI-D patients who required admission to the ICU and vasopressor support had high mortality rates, reaching up to 50%. However, 55% to 80% of survivors experienced full recovery of kidney function at hospital discharge, and only 5% of survivors progressed to ESKD.^16–18^ In our study, we found that ICU admission and vasopressor use were significantly higher in recovered patients compared to non-recovered patients (60% vs. 30%; *P* = 0.03) and (50% vs. 9%; *P* < 0.001), respectively. There are several explanations for such findings. First, most AKIs in critically ill patients are presumed to be due to ATI, which does not usually lead to ESKD.^19,20^ Second, ICU patients often receive more intensified promoting renal recovery measures, such as hemodynamics optimization, blood pressure support use, and judicious ultrafiltration during RRT, especially since avoiding hypotension episodes during intermittent dialysis is crucial in promoting kidney function recovery.^11,12,21,22^ Third, early recognition of AKI and prompt treatment in an ICU setting might help minimize kidney injury.^23^

Whether frequent hemodialysis for AKI-D patients can lead to improvement in renal recovery is still controversial. Schiffl et al. found that daily dialysis resulted in fewer hypotensive episodes during hemodialysis, more rapid resolution of AKI, and a lower mortality rate than less frequent dialysis.^24^ On the other hand, Vijayan et al. found that the likelihood of renal recovery was lower in patients who received hemodialysis 6 days per week than those who received hemodialysis thrice weekly.^25^ Another study showed that intensified dialysis was associated with a significant reduction in urine output,^26^ despite the hypothesis that intensified dialysis might result in a lower rate of ultrafiltration and less frequency of hypotensive episodes. However, there was no difference regarding renal recovery between intensified and standard intermittent dialysis in another trial.^27^ Our study found a non-significant effect of the number of hemodialysis sessions during hospitalization on renal recovery in multivariate analysis, although we noticed that recovered patients received more dialysis sessions during hospitalization than non-recovered patients (18.4 ± 17.4 vs. 10.2 ± 5.9; *P* = 0.006) which may be explained by more extended hospital stay in the recovered patients than non-recovered patients (39.0 ± 33.7 vs 20.8 ± 13.3; *P* = 0.003).

**Figure 2:**
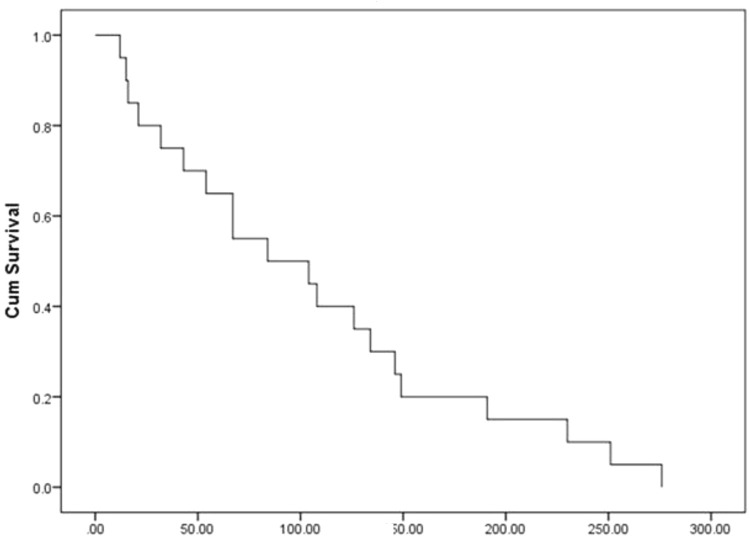
Time to recover in days.

Recovery from ESKD is frequently reported and detected in 0.3% to 8% of chronic dialysis patients, especially during the first year of dialysis.^28^ AKI accounts for most cases of ESKD recovery, even when CKD progression is the cause of ESRD.^29^ In a recent meta-analysis of almost 2.5 million chronic dialysis patients, the prevalence of renal function recovery was 1.5% of patients. The average duration of dialysis discontinuation was 10 months after starting treatment.^30^ For this reason, unlike previous studies designed to follow the patients for 3 months,^10–14^ we extended follow-up for AKI-D patients who still require dialysis after hospitalization to 6 months before being declared ESKD. Our patients’ average time to renal recovery was 93 ± 61 days, and 50% of AKI-D patients recovered after more than 3 months from hemodialysis initiation (Figure 2). Interestingly, we found that 20% of patients with baseline eGFR < 15 mL/min/1.73 m^2^ had renal recovery, highlighting the possibility of renal recovery even in patients with advanced CKD.

Severe AKI requiring dialysis might result in significant ultrastructural changes in renal pathology, which ultimately lead to worsening of kidney function and, consequently, CKD either de novo or worsening of pre-existing CKD.^31^ Few trials concluded that regular follow-up of recovered AKI-D patients is associated with a reduction in mortality rate.^32,33^ Recovered patients in our study continued to be followed at outpatient nephrology and low-clearance clinics for an average of 17.6 months. Twenty-five percent of recovered patients (*n* = 5) returned to dialysis after 6 to 44 months, and 35% died after 5 to 35 months from renal recovery. Currently, there are no standard guidelines that regulate the follow-up of AKI-D patients after recovery of renal function.

Our study has several strengths. First, the electronic medical record system used in our hospital is the same system used in all dialysis centers and outpatient clinics, which facilitates patient follow-up and ensures accurate data collection. Second, most patients in our study were from the Middle East, a population that has not been extensively studied in this context. Last, unlike previous studies, we continued to follow patients after renal recovery, providing long-term outcome data for recovered AKI-D patients.

This study also has several limitations. First, it was a retrospective, single-center study, which may limit the generalizability of the findings. Second, the sample size was relatively small, which may have reduced the power to detect all potential predictors of renal recovery. Third, COVID-19 was not included as a potential cause of AKI or as a variable in outcome analyses, as data collection was initiated before the onset of the pandemic. Finally, all patients received hemodialysis; none had peritoneal dialysis or continuous RRT. As a result, we were unable to assess the impact of vasopressor use across different dialysis modalities.

## 5. CONCLUSION

Renal recovery after discharge can occur in up to one-third of patients with AKI-D, including those who remain dialysis-dependent for more than 3 months. Vasopressor use during hospitalization was associated with a higher likelihood of renal recovery; however, this observation needs to be confirmed in prospective, multicenter studies. Close post-discharge monitoring of AKI-D patients is warranted, and evidence-based guidelines are needed to optimize their management.

## ETHICAL APPROVAL

Approved by the Medical Research Center and the institutional review board at Hamad Medical Corporation, Qatar (MRC-01-22-749).

## Figures and Tables

**Supplementary Table 1.** Types and duration of vasopressors in recovered and non-recovered AKI-D patients.

Patient Number	Group	Vasopressors type	Duration
1	Recovered	Dopamine	5 days
2	Recovered	Dobutamine Dopamine	4 days 7 days
3	Recovered	Dopamine	2 days
4	Recovered	Dopamine	2 days
5	Recovered	Noradrenaline	12 days
6	Recovered	Phenylephrine Noradrenaline Adrenaline	1 day 3 days 1 day
7	Recovered	Adrenaline Dopamine	2 days 5 days
8	Recovered	Adrenaline Dobutamine Noradrenaline	1 day 3 days 3 days
9	Recovered	Dobutamine Dopamine	7 days 1 Day
10	Recovered	Dopamine Noradrenaline	1 day 5 days
11	Non-recovered	Noradrenaline	18 days
12	Non-recovered	Noradrenaline	5 days
13	Non-recovered	Noradrenaline	9 days
14	Non-recovered	Noradrenaline Dopamine	2 days 1 Day

**Supplementary Table 2.** Long-term outcomes of recovered AKI-D patients.

Patient number	Clinical outcome	Time to clinical outcome	eGFR at clinical outcome (ml/min/1.73 m^2^)
1	Death	5 months	23
2	Death	6 months	23
3	Death	7 months	12
4	Death	7 months	28
5	Death	16 months	12
6	Death	28 months	43
7	Death	35 months	34
8	Back to HD	6 months	11
9	Back to HD	11 months	8
10	Back to HD	12 months	13
11	Back to HD	21 months	10
12	Back to HD	44 months	15
13	Kidney transplant	7 months	15
14	Following in clinic	5 months	55
15	Following in clinic	6 months	20
16	Following in Clinic	49 months	9
17	Left the country	7 months	27
18	Left the country	13 months	14
19	Left the country	23 months	36
20	Left the country	44 months	22

**Table 1.** Baseline characteristics of the study population.

Characteristics	Study population (*n* = 64)
Age, years, mean ± SD	64.3 ± 15.3
Gender, *n* (%):	
Male	29 (45.3)
Female	35 (54.6)
Ethnic group, *n* (%)	
Middle Eastern	61 (95.3)
Others	3 (4.7)
Comorbidities, *n* (%)	
Diabetes	52 (81.3)
Hypertension	56 (87.5)
Congestive heart failure	28 (43.8)
Coronary artery disease	26 (40.6)
Peripheral vascular disease	2 (3.1)
Chronic kidney disease	55 (85.9)
Chronic liver disease	2 (3.1)
Active malignancy	1 (1.6)
Baseline serum creatinine, μmol/L, mean ± SD	271.2 ± 138.9
Baseline eGFR, mL/min/1.73 m^2^, mean ± SD	29.4 ± 26
Baseline eGFR, mL/min/1.73 m^2^, *n* (%)	
≥60	7 (10.9)
30–59	11 (17.2)
15–29	25 (39.1)
<15	19 (29.7)
Unknown	2 (3.1)
Serum creatinine on admission, μmol/L, mean ± SD	583.6 ± 238.4
Causes of AKI, *n* (%):	
Acute tubular injury	32 (50)
Cardiorenal syndrome	12 (18.8)
Glomerulonephritis	2 (3.1)
Obstructive uropathy	2 (3.1)
Others	9 (14.1)
Unknown	7 (9.7)
ICU admission, *n* (%)	25 (39.1)
ICU stay, days, mean ± SD	13.5 ± 9.7
Vasopressors use, *n* (%)	14 (21.9)
Mechanical ventilation, *n* (%)	10 (15.6)
Dialysis details	
Days on RRT during hospitalization, *n* (%)	25.9 ± 23.0
Number of hemodialysis sessions during hospitalization, *n* (%)	12.5 ± 11.4
Weekly hemodialysis sessions during hospitalization, mean ± SD	3.8 ± 1.27
Dialysis access on discharge, *n* (%):	
Arteriovenous fistula	4 (6.3)
Central venous catheter	60 (93.8)

**Table 2.** Clinical outcomes of the study population.

Outcome	Study population (*n* = 64)
Renal outcomes at 6 months, *n* (%)	
Renal recovery	20 (31.3)
Dialysis-dependent	44 (68.7)
Renal recovery in 6 months based on baseline eGFR, mL/min/1.73 m^2^, *n* (%)	
≥60	4 (57)
30–59	5 (45)
15–29	6 (24)
<15	4 (21)
Unknown	1 (50)
Time to renal recovery, *n* (%):	
<1 month	4 (20)
1–3 months	6 (30)
3–6 months	10 (50)
Time to renal recovery, days, mean ± SD	93 ± 61
Hospital readmission, *n* (%)	27 (42.2)

**Table 3.** Univariate analysis of variables associated with renal recovery in patients with AKI-D.

Variable	Recovered (*n* = 20)	Non-recovered (*n* = 44)	*P* value
Age, years, mean ± SD	66.4 ± 11.6	63.0 ± 17.0	0.42
Male gender, *n* (%)	11 (55)	18 (41)	0.42
Non-Middle Eastern race, *n* (%)	**3 (15)**	**0 (0)**	**0.03**
Comorbidities, *n* (%)			
Diabetes	16 (80)	36 (86)	1.00
Hypertension	18 (90	38 (86)	1.00
Congestive heart failure	10 (50)	18 (41)	0.59
Coronary artery disease	9 (45)	17 (39)	0.78
Peripheral vascular disease	1 (5)	1 (2.2)	0.53
Chronic kidney disease	17 (85)	40 (91)	0.67
Liver cirrhosis	1 (5)	1 (2)	0.53
Malignancy	0	1 (2)	1.00
AKI cause, *n* (%)			
Acute tubular injury	10 (50)	22 (50)	
Glomerulonephritis	0	2 (5)	
Obstructive uropathy	1 (5)	1 (2)	0.73
Cardiorenal	5 (25)	7 (16)	
Others	2 (10)	7 (16)	
Unknown	2 (10)	5 (11)	
Baseline serum creatinine, μmol/L, mean ± SD	**210.1 ± 128.7**	**293.1 ± 139.6**	**0.03**
Baseline eGFR, mL/min/1.73 m^2^, mean ± SD	38.4 ± 25.8	26.9 ± 27.1	0.13
Baseline eGFR, mL/min/1.73 m^2^, *n* (%):			
≥60	4 (20)	3 (7)	
30–59	5 (25)	6 (14)	
15–29	6 (30)	19 (43)	0.19
<15	4 (20)	15 (34)	
Unknown	1 (5)	1 (2)	
Serum creatinine at hemodialysis initiation, μmol/L, mean ± SD	518.6 ± 270.2	616.1 ± 231.3	0.14
Blood urea nitrogen at hemodialysis initiation, mmol/L, mean ± SD	38.4 ± 31.0	36.4 ± 11.5	0.71
ICU Admission, *n* (%)	**12 (60)**	**13 (30)**	**0.03**
Mechanical ventilation, *n* (%)	5 (25)	5 (11)	0.26
Vasopressors use, *n* (%)	**10 (50)**	**4 (9)**	**<0.001**
Intradialytic hypotension, *n* (%)	2 (10)	11 (25)	0.20
Number of hemodialysis sessions during admission, mean ± SD	**18.4 ± 17.4**	**10.2 ± 5.9**	**0.006**
Length of hospital stay, days, mean ± SD	**39.0 ± 33.7**	**20.8 ± 13.3**	**0.003**
Dialysis access on discharge			
Central catheter, *n* (%)	19 (95)	41 (93)	
Arteriovenous fistula, *n* (%)	1 (5)	3 (7)	1.00
Laboratory results at discharge
Creatinine at discharge, μmol/L, mean ± SD	322.5 ± 182.8	406.4 ± 160.4	0.07
Urea Nitrogen at discharge, mmol/L, mean ± SD	15.0 ± 10.1	16.3 ± 7.7	0.56
Albumin at discharge, g/L, mean ± SD	25.9 ± 5.6	26.7 ± 6.0	0.60
Hemoglobin at discharge, g/dL, mean ± SD	9.2 ± 0.9	9.4 ± 1.5	0.64
Average weekly predialysis creatinine, μmol/L, mean ± SD	**334.0 ± 162.7**	**491.8 ± 186.2**	**0.002**
Hospital readmission, *n* (%)	8 (40)	19 (43.1)	1.00

Bold values indicate the significant predictors of renal recovery.

**Table 4.** Multivariate analysis of variables associated with renal recovery.

Predictors	Recovered (*N* = 20)	Non-recovered (*N* = 44)	Odds ratio	95%CI	*P* value
Vasopressors use	**10 (50)**	**4 (9)**	**16.244**	**1.22–217.17**	**0.035**
Average weekly predialysis creatinine, μmol/L	334.0 ± 162.7	491.8 ± 186.2	1.006	1.00–1.01	0.058
Baseline serum creatinine, μmol/L	210.1 ± 128.7	293.1 ± 139.6	1.00	0.99–1.01	0.78
ICU admission	12 (60)	13 (29.5)	0.19	0.02–2.40	0.31
Dialysis sessions during admission	18.4 ± 17.4	10.2 ± 5.9	0.96	0.86–1.07	0.38
Non-Middle Eastern race	3 (15)	0 (0)	0.00	0.00	1.00

Bold values indicate the significant predictors of renal recovery.

**Table 5.** Comparison of vasopressor use between recovered and non-recovered AKI-D patients.

Variable	Recovered (*n* = 10)	Non-recovered (*n* = 4)	*P* value
Dopamine, *n* (%)	7 (70)	1 (25)	0.24
Norepinephrine, *n* (%)	4 (40)	4 (100)	0.085
>1 vasopressor, *n* (%)	6 (60)	1 (25)	0.56
Duration of vasopressors, mean (± SD)	3.6 ± 2.9	7.0 ± 6.9	0.11
